# Image analysis procedure for studying Back-Diffusion phenomena from low-permeability layers in laboratory tests

**DOI:** 10.1038/srep30400

**Published:** 2016-07-28

**Authors:** Fabio Tatti, Marco Petrangeli Papini, Massimo Raboni, Paolo Viotti

**Affiliations:** 1 Department of Civil, Building and Environmental Engineering (DICEA), University of Rome “La Sapienza”, Via Eudossiana 18, 00184, Rome, Italy; 2 Department of Chemistry, University of Rome “La Sapienza”, Piazzale Aldo Moro 5, 00185, Rome, Italy; 3 School of Industrial Engineering, University LIUC-Cattaneo, Corso Matteotti 22, I-21053, Castellanza, VA, Italy

## Abstract

In this study, the long-term tailing derived from the storage process of contaminants in low-permeability zones is investigated. The release from these areas in the groundwater can be considered a long-term source that often undermines remediation efforts. An Image Analysis technique is used to analyze the process and evaluate the concentrations of a tracer at different points of the test section. Furthermore, the diffusive flux from the low-permeability lenses is determined. To validate the proposed technique, the results are compared with samples, and the diffusive fluxes resulting from the low-permeability zones of the reconstructed aquifer are compared with a theoretical approach.

Remediation efforts during past decades have often shown their limits in the process of restoring groundwater quality when non-aqueous phase liquid (Dense Non-Aqueous Phase Liquid-DNAPL or Light Non-Aqueous Phase Liquid-LNAPL) contamination is encountered. These contamination scenarios are recognized as becoming source zones that cause long-term plumes that are very difficult to remove completely or isolate. A similar situation can occur if the plume encounters low-permeability zones during its flow. In this case, these zones can be saturated from the contaminant and became future contamination sources characterized by long-term releasing contaminant tails, as in the case of NAPL. In fact, low-permeability zones appear to be able to store relatively high contaminant concentrations deep within their structure. The main process seems to be dominated by molecular diffusion, and the concentration gradient between low- and high-permeability zones determines the flux of dissolved substance into the lower permeability lenses (Forward Diffusion). The situation persists until the end of the passage of the plume; subsequently, there is an inversion of the gradient direction that leads to a slow re-distribution of the contaminant from the lower permeability zones back to the higher permeability zones (Back Diffusion). Therefore, the “Back Diffusion” process is expected to occur when the contaminant flux from a source zone that passes directly into the ground waters is strongly reduced or reduced to almost zero. This aspect can indicate the similarity of behavior between this type of source and others that have as a fundamental characteristic a long-lasting release, such as those derived from NAPL contamination (DNAPL and LNAPL). The main difference can be observed in that the separated phases release directly in the flow from their pooled or residual status; thus, the flux is mostly dependent, as is known, on the solubility of the substance/substances in the solvent phase (Falta *et al*.^1^, Seyedabbasi *et al*.^2^). In contrast, in the examined cases, the driving force of the release is the diffusion that is governed from gradient, and the resulting flux is thus influenced by mechanical parameters such as the grain size and molecular diffusion (Rolle *et al*.^3^). Aspects to be defined are the importance of the different elements at the boundary in the process.

First, studies regarding Back Diffusion appeared during groundwater restoration performed by Liu and Ball^4^, Chapman *et al*.^5^ and Parker *et al*.^6^. In these papers, Back Diffusion was demonstrated by field measurements after the hydraulic isolation of the primary source. Based on these studies, researchers focused their attention on low-permeability zones that can be viewed as a secondary source. Due to the difficulty of collecting data in the field, studies on Back Diffusion were performed mainly at the laboratory scale (Sale *et al*.^7^ Chapman *et al*.^8^, Wilking *et al*.^9^, Yang *et al*.^10^). In these experiments, aquifer structures were reconstructed in tanks with thin clay lenses or with aquitard layers at the bottom. In most of the laboratory experiments, tracers were realized, with the aim of observing the processes of storage and release of contaminant from low-permeability zones, paying particular attention to the consequent possible plume tailing. In recent years, several laboratory experiments were coupled to Image Analysis (I.A.) techniques that permit, through the use of specific procedures, the estimation of the contaminant concentration in the test section without the use of invasive instruments (Huang *et al*.^11^, Theodoropoulou *et al*.^12^, McNeil *et al*.^13^, Werth *et al*.^14^, Wilking *et al*.^9^, Yang *et al*.^10^). Different I.A. methods can be used to measure the tracer concentrations: they can be roughly re-conducted according to the light transmission technique (Zinn *et al*.^15^, Catania *et al*.^16^, Konz *et al*.^17^, Wang *et al*.^18^, Jaeger *et al*.^19^) or the reflection light technique (Konz *et al*.^20^). The main objective of the techniques used in laboratory tests is to investigate the Back Diffusion phenomenon while improving the practical approaches and numerical models used for its management during restoration treatments. For this reason, in several reported studies, different types of numerical models were validated using experimental results from laboratory tests. The numerical approaches range from analytical solutions for the simplest scenarios (Sale *et al*.^7^, Brown *et al*.^21^ and Yang *et al*.^10^,^22^) to numerical solutions based on finite-element techniques (Chapman and Parker^5^, Parker *et al*.^6^, Chapman *et al*.^8^) or finite-difference techniques (Chapman *et al*.^8^) for more complicated situations.

For these reasons, we focused on realizing the experimental tests on the Back Diffusion process from the low-permeability lenses described in this paper. An I.A. technique is used to evaluate the concentrations of a tracer at different points of the test section. Briefly, the test procedure was conducted using periods of flushing and periods of rest. At night, the system was left in a rest situation, thus allowing the maximum diffusive effects from the low permeability lenses; during the day, the system was subject to flushing. Images (Red Green Blue–RGB format) were acquired during the different phases. The tracer concentrations in both the effluent from the tank and the medium are calculated, and the diffusive flux from the low permeability lenses is determined. To validate the proposed I.A. technique, the concentration values in effluent estimated by I.A. are compared with the effluent concentration determined by fluorometer analysis. Furthermore, the calculated diffusive fluxes from the low-permeability parts of the aquifer, as reconstructed by I.A. are compared with those derived from the theoretical approach proposed by Yang *et al*.^10^.

## Methods

### Experimental setup

**Figure 1:**
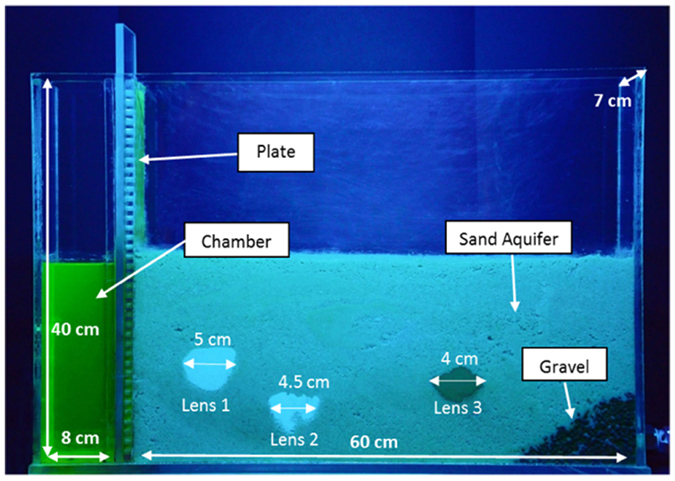
The apparatus used for the experiment.

The representation of the Back Diffusion phenomenon was conducted at laboratory scale using a Plexiglas tank with dimensions of 68 cm (horizontal length) × 40 cm (height) × 7 cm (depth) (Fig. 1) divided into two parts. The first chamber was used to control the water level and limit the turbulence effects, and the second was filled with porous media. The two resulting chambers were connected by a punched plate to allow water flow. At the end of the tank was inserted a valve for managing the hydraulic gradient that controlled the flow. To prevent sand escape, a gravel layer was located before the outlet. Two peristaltic pumps connected to the first chamber and to the tap outlet were used to maintain a constant flow rate during the experiment.

The model roughly reproducing the aquifer consists of a high-permeability layer in which some low permeability lenses are inserted. The high-permeability layer was realized using quartz sand. To reproduce low-permeability zones, three lenses of different form and grain size were inserted. The first two lenses were composed of the quartz flour (Silverbond SA 12S and Silverbond SA 4K), and the third lens was composed of sodium bentonite. The quartz flours were chosen to simulate the low-permeability media because of their insolubility in water (rather than reactivity) and their light color; this last property allows the diffusion of a colored tracer to be observed clearly, even inside. For each material laboratory, measurements were performed to estimate the particle size and porosity. The particle size analyses of quartz flours and sodium bentonite were performed using a Sedigraph. The method of measurement is based both on Stokes’ law and on the absorption of X-rays as functions of the concentration of sediment in the liquid dispersant and the absorption spectrum of the dispersing liquid used (baseline). A deconvolution algorithm of the absorption spectra allows, by subtracting the absorption spectrum of the baseline from the measures, the particle size analysis to be obtained in terms of the size of the grains vs percentage by weight. As shown in Table 1, sodium bentonite has a medium grain size (D_50_) that is an order of magnitude smaller than those of lenses 1 and 2, which have D_50_ values of 15 μm and 12 μm, respectively. The particle size analysis of quartz sand was performed by sieving, according to the ASTM standards (ASTM^23^). The medium grain size of the sand was estimated to be 700 μm (Table 1). To determine the porosity of sand, quartz flours and sodium bentonite, the following relation was used:


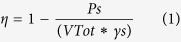


where Ps is the dry weight of the material, VTot is the sample volume and γs is the density of the material. To estimate the value of sand Ps, the Sand-Cone Method was used, according to the ASTM standards (ASTM^23^). To determine the sodium bentonite Ps, three samples with known volumes were filled with the saturated material and then weighed. The samples were dried at a temperature of 110 °C for 24 hours and were weighed again, thus allowing their water moisture and Ps to be estimated. The same laboratory procedure was used to determine the value of the quartz flours’ Ps. Table 1 presents the porosity value for each material obtained from the average of the three samples’ values.

**Figure 2:**
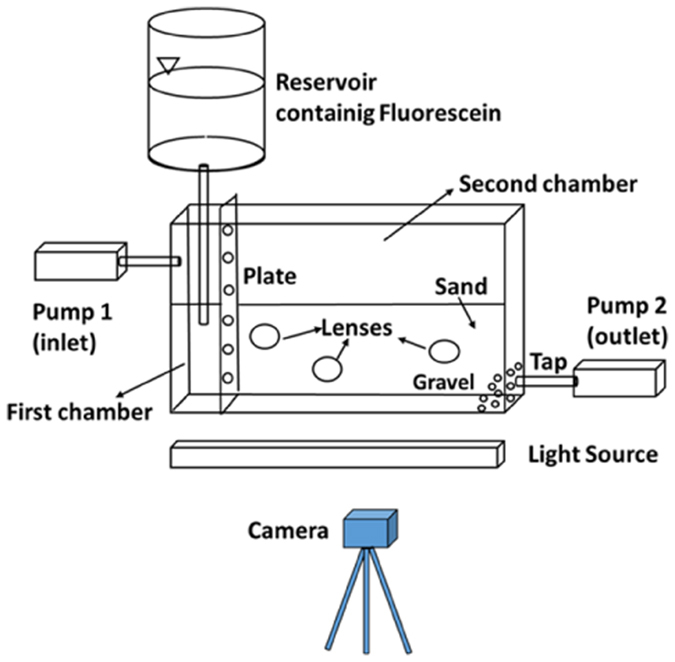
Experimental set-up.

Sodium fluorescein was used as a colored tracer. Fluorescein is a non-toxic and non-reactive compound. It was chosen because it emits a light that varies from green to orange as a function of its concentration when it is excited by UV light (Sabatini and Austinal^24^). In this way, the tracer results are clearly visible in porous media, thus allowing the application of I.A. In the experiment described in this paper, the fluorescence was stimulated using an ultraviolet light bulb (Philips Actinic BL TL-D 18W-10 UV-A G13). The entire experimental apparatus is shown in Fig. 2 and consisted of the previously described tank with the inlet and outlet systems (two peristaltic pumps), a reservoir for the fluorescein, a UV lamp positioned in front of the tank and a 3CCD camera. The experimental procedure used I.A. to analyze the redistribution process using the small grain size lenses.

### Test procedure

**Figure 3:**
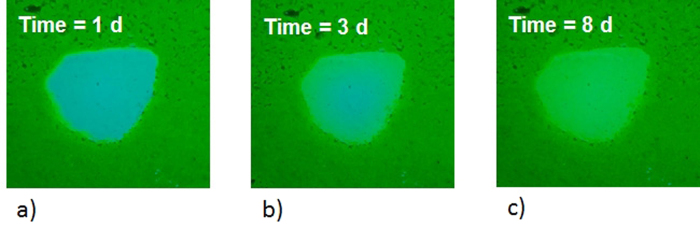
Fluorescein diffusion in lens 1 during the saturation phase.

**Figure 4:**
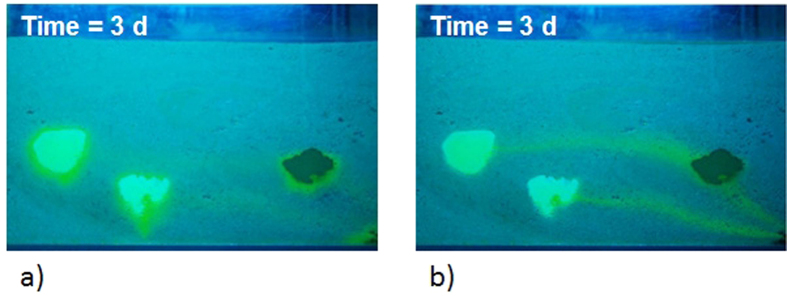
The Back Diffusion Phenomenon observed in the tank. Fluorescein released from lenses before water flushing in the third day (**a**) and during water flushing in the third day (**b**).

In the first step of the experiment, the tank was saturated with water. The experiment was performed using a constant flow rate of approximately 26 mL/min. Subsequently, a solution containing 2 g/L of dissolved fluorescein was slowly injected into the first chamber. The concentration of the solution was monitored over time using samples that were collected at the outlet. The sodium fluorescein concentration in every sample was estimated by measuring the intensity of the tracer fluorescence. Each water sample was excited by UV light at 254 nm, and the intensity of the emitted light (fluorescence) by fluorescein was measured using a fluorometer. Based on the fluorescence intensity of samples with known tracer concentrations, a relationship was determined between the intensity of fluorescence and the fluorescein concentration. When the sample concentration reached a plateau of 2 g/L, this concentration was considered to exist in the whole tank. At that time, the injection of the fluorescein solution in the first chamber was stopped, the top of the tank was sealed to avoid evaporation and the second chamber was kept in saturated condition for three weeks. During that time, fluorescein diffusion advanced visibly in the lenses (Fig. 3). At the end of this phase, water was flushed for 14 days. For the first 5 days, two pore volumes were flushed daily, and for the remaining 9 days, only one pore volume of water (approximately four liters) was flushed. The daily flushing phase was conducted during the daylight, and during the night, the flow was stopped and then re-started on the following morning. The flushing of one pore volume lasted approximately three hours; therefore, the daily flushing phase continued for approximately 6 hours on the first 5 days and approximately 3 hours on the remaining 9 days. The diffusion phenomenon from the low-permeability zones was clearly visible, as shown in Fig. 4a, which clearly shows the solute that was released from the low-permeability lenses during the night phase and redistributed via diffusion in the higher-permeability layer. Figure 4b shows the tails of the released dye during water flushing. Moreover, a lowering of diffusive flow over time and, subsequently, of the tails in the sand was observed. During the flushing, the tail of the first lens reaches the third lens. This fact is negligible for our purposes because we use I.A. to estimate both the effluent concentration (which is the sum of the three tails) and the diffusive flux from the lenses during the no-flushing phase.

### Image Analysis

**Figure 5:**
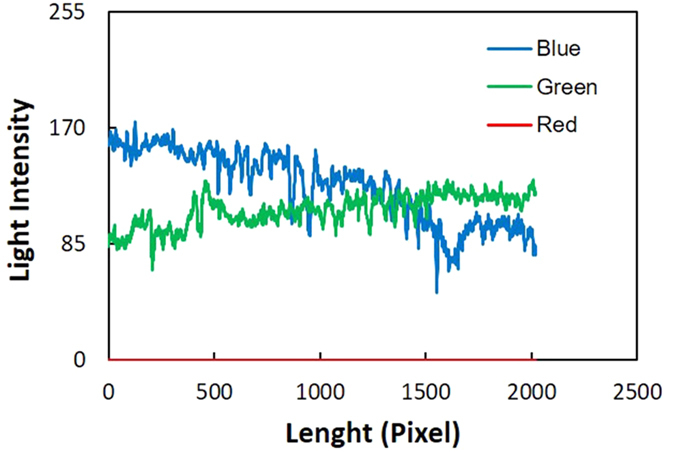
Light intensity in a section of the tank.

To determine the fluorescein concentration released by the low-permeability lenses, an I.A. technique based on the intensity of the light emitted by the tracer was used. The source of UV light placed in front of the tank excites the dissolved fluorescein, which subsequently reemits light. Images of the diffused tracer were acquired using a CCD camera; to increase the clarity of the images, a UV filter (Hoya Pro1 Digital Filter UV) was applied on the camera lens. The small thickness of the tank compared with the other dimensions allowed a 2D approach to be used for the analysis, considering the dye distribution to be uniform in the third dimension. The analysis was performed with two goals: the first was to evaluate the trend of effluent concentration, and the second was to derive diffusive flow values from the low-permeability lenses. To achieve these goals, a calibration procedure was conducted using an additional small Plexiglas tank filled with quartz sand and saturated with solutions at different known concentrations of fluorescein. Due to the phenomenon of brightness attenuation far from the center area of the image (M. Yang *et al*.^10^), the behavior for each color was investigated, locating the calibration tank once at the center part of the test section and once relatively close to the outlet. In both positions, images of the small tank containing solutions were collected at known tracer concentrations. Each pixel of the pictures displays a color due to the combination of Red, Green and Blue (RGB color system). It is possible to divide the three colors channels to perform a transformation on each picture, resulting in three different images in grey scale. In this manner, each pixel can assume integer values from 0 (Black) to 255 (White), thereby enabling the measurement of the pixel intensity of the whole image in each channel. The collected pictures of the small tank were elaborated using the above-mentioned I.A. procedure. The three colors channels were separated: Red values resulted in negligible intensity, and the intensities of the Blue and Green light were measured (Fig. 5).

**Figure 6:**
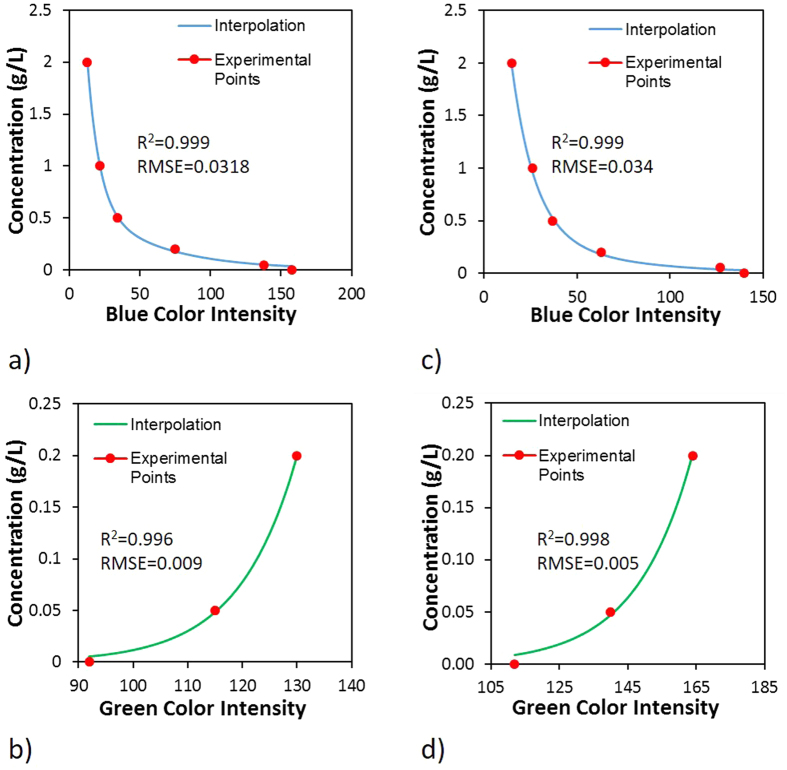
Calibration results. Relations between color intensity and fluorescein concentration in middle section of the tank (**a,b**) and the inside part of the tank (**d,c**).

The color intensity values obtained by image elaboration matched to the respective concentrations were interpolated to determine the analytical relations between the fluorescein concentration and the Green and Blue intensities. The relations were found to be nonlinear at both of the positions used for calibration. A polynomial relation based on exponential functions is found for Blue color (Fig. 6a,c); this result is in agreement with studies by Yang *et al*.^10^, Catania *et al*.^16^, Konz *et al*.^17^ and Jones and Smith^25^. Conversely, a simple exponential function fits the relation between the Green color intensity and the concentrations (Fig. 6b,d) for both positions. This result is valid for concentrations lower than 0.2 g/L because for higher concentration values, an over-saturation intensity is observed (Konz *et al*.^17^). The relations obtained appear to fit the experimental points with high precision. This result is demonstrated by the high value assumed from the correlation coefficient (R^2^) obtained and the low values of the Root Mean Square Error (RMSE). R^2^ is greater than 0.99 for both the positions analyzed, and these values are similar for the Blue and Green colors. The RMSE was evaluated to be on the order of 10^−2^ for Blue functions and 10^−3^ for Green functions. The agreement between the experimental points and the interpolating relations is an important result for successfully estimating the effluent concentration and the diffusive flux using I.A.

## Results

### Determination of effluent concentrations

**Figure 7:**
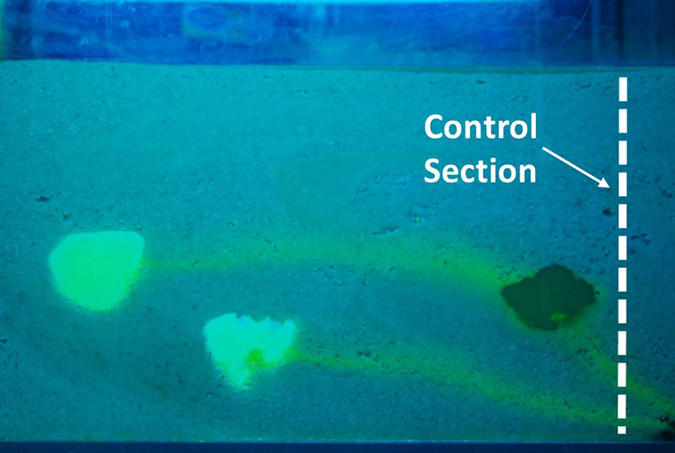
The control section utilized to evaluate the fluorescein concentrations by I.A.

The trend of effluent concentrations was then obtained using the aforementioned I.A. procedure. During the flushing phase of the experiment, pictures of the tank were collected; in each picture, the Blue and Green color channels were separated and converted into gray scale images. The concentrations were evaluated at the control section in the side part of the tank, as reported in Fig. 7. In each image, the average color value of the control section calculated by the I.A. was considered; next, using the functions obtained during the calibration phase, a concentration was assigned to that value. The evaluated concentrations based on the proposed procedure were validated by comparing the obtained concentration values with those of fluorescein in the effluent samples. During the flushing phase for the first 5 days, samples containing 50 mL of water were collected, and their sodium fluorescein concentrations were estimated using the procedure described in this paper. The results allow the different reliability levels of both Green and Blue functions to be validated, depending on the concentration values.

**Figure 8:**
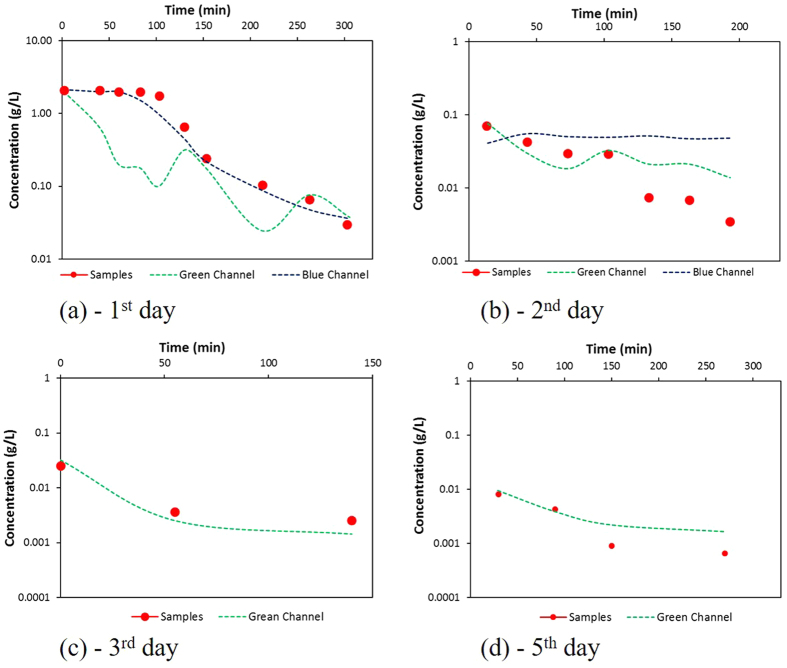
Comparison between the measured concentrations and those obtained from I.A.

An error value (E) was used to evaluate the fitness level between the obtained and measured data (Yang *et al*.^10^):


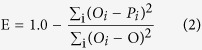


where P_i_ is the calculated concentrations by I.A., O_i_ is the measured concentrations and O is the average of the sample concentrations. The E values range from negative infinity to 1.0, with values closer to 1.0 indicating a better fit. The E values obtained ranged from 0.9 to 0.3 (Table 2). The obtained values were considered suitable for demonstrating the efficiency of the proposed procedure. During the first day, the concentration values of effluent reached 0.1 g/L, and the Blue-function could be considered more suitable (Fig. 8a). Indeed, the value of E for Blue color was 0.9, which was substantially larger than the value obtained for the Green function, i.e., −0.4 (Table 2). On the second day, the trend changed drastically: the concentrations were lower than 0.1 g/l, and the E value of Green increased, becoming larger than the Blue one. This observation suggested that the Green function can be used to describe the low concentration range. The decreased concentration value in the following days led to the use of only the Green function to estimate the trend of effluent concentration during this period (Fig. 8c,d). The lowest E value was obtained on the fifth day; this value resulted from the low concentration values of the fluorescein, which determined the larger errors during the I.A. procedure. Difficulties in obtaining a constant light source caused variations in light intensity in the acquired images, which resulted in small fluctuations in the fluorescein concentrations (Yang *et al*.^10^, Konz *et al*.^17^). However, their presence did not spoil the results; as a result, Fig. 8 shows the concentration trends after a smoothing procedure was applied.

### Determination of Diffusive Flux

**Figure 9:**
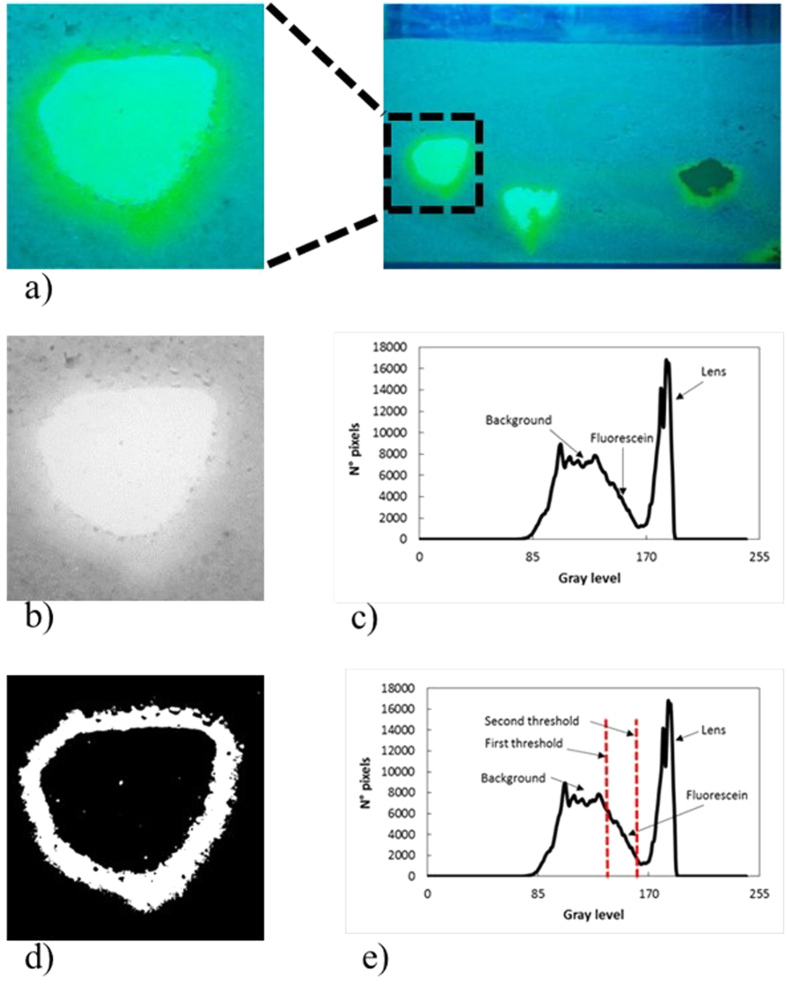
The I.A. procedure used to evaluate the diffusive flux. The selected area around the first lens (**a**). The same area converted into a gray scale image (**b**) and its histogram of pixel frequencies (**c**). The same area after the application of the two thresholds (**d**) and in (**e**), the histogram of the selected Gray levels used for filtering the image.

To estimate the mean diffusive flow of the tracer released from low permeability lenses, the image captured each day before the beginning of the flushing phase was analyzed. A defined area of interest around each lens on every picture (Fig. 9a) was examined using the I.A. procedure, resulting in a gray scale image (Fig. 9b). The histogram representing the distribution of pixels for each gray level reveals two distinct peaks that represent the light intensity corresponding to the presence of lens or of sand (background). The values between the peaks represent the presence of fluorescein (Fig. 9c). Two thresholds were applied to account for the background and the lens (Fig. 9d,e). The number of pixels for each value of gray level contained in the selected range was determined using a specific I.A. program. Each group of pixels characterized by a specific gray level was associated with a value of the fluorescein concentration (as determined using the relations between the fluorescein concentrations and light intensity) and a value of the area. The area represented by each pixel was equal to 2.83 × 10^−5^ cm^2^. The procedure provides the total mass of fluorescein released from each lens. It is assumed that the molecular flux from the lenses can be considered negligible during the flushing phase. This can be assured by the values of the Peclet Number (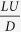), defined as U (Effective velocity) multiplied by L (reference length) and divided by D (molecular diffusivity of the fluorescein). High Peclet Number values (Pe ≫ 1) prove that the molecular diffusion transport is negligible compared to advective transport, whereas low values (Pe ≪ 1) prove the inverse. In our test, the Peclet Number assumes values ranging from 46 to 4600, depending on the value chosen for L. For example, using D = 13 × 10^−10^ m^2^/s and U = 10 m/d, for L (lens diameter) = 4 cm, we obtain Pe = 4600; alternatively, for L (d_50_ of the low permeability medium) = 4 × 10^−3^ cm, we obtain Pe = 46.

Using the above-mentioned hypothesis and dividing by both the perimeter of the lens and the non-flushing time, the values represent a diffusion flow per unit of time and per unit of length for every lens.

**Figure 10:**
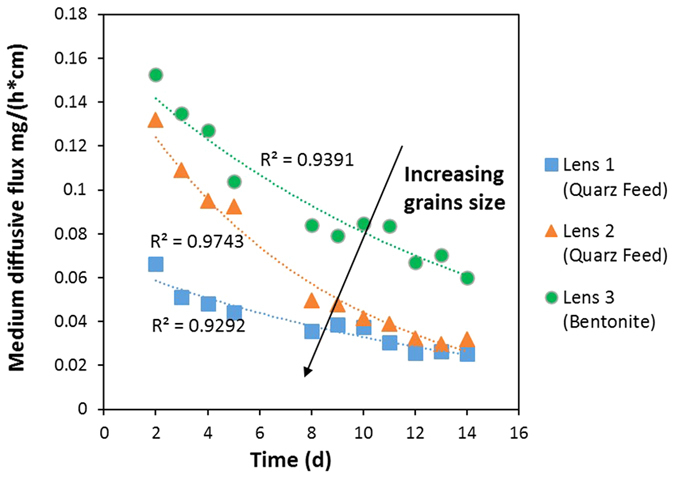
Medium diffusive flow trends vs time obtained using I.A.

The diffusive flow was calculated for each lens for 13 days (from 2nd until 14th), and its trend as a function of time is shown in Fig. 10. It can be assumed that for all lenses, the intensity of diffusive flow presents an exponential decay in time. The exponential function fits the data by I.A., with R^2^ ranging from 0.92 to 0.97 for all lenses. The experimental trends clearly show an increase in medium diffusive flux from lens 1 to lens 3. This result can be directly linked to the grain size: the smaller the grain size is (in our experiment, d_50_ = 1.4 μm), the larger their diffusive flux is, presumably because of a greater storage capacity in the smaller medium grain size. Thus, according to Fig. 10, lens 3 is found to be able to store more diluted substance; as a result, its release is larger than that of the others. These results can be used to further confirm that low-permeability zones must be considered as a long-lasting contaminant source, recalling the behavior of other similar sources such as DNAPL and LNAPL.

**Figure 11:**
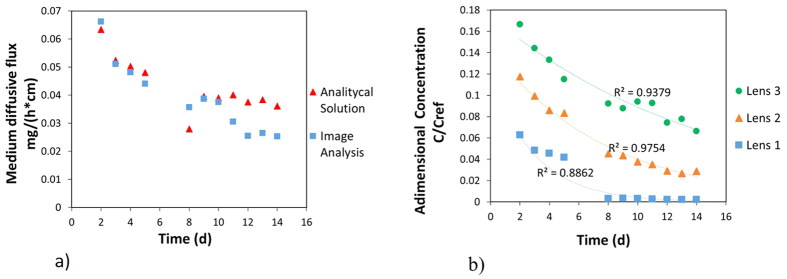
Comparison between the experimental and theoretical results. Comparison between the medium diffusive flux obtained using I.A. and the analytical solution proposed by Yang *et al*.^10^ (**a**). Comparison between the concentrations derived from the I.A. procedure for the three lenses (**b**).

Lens 1, because of its circular shape, was used to compare the results with those obtained by means of the analytical solution of diffusive flux proposed by Yang *et al*.^10^.


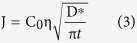


where J is the diffusive flux, C_0_ is the solute constant concentration released into the sand, η is the porosity of low permeability zone (Table 1) and t is the time of diffusive flow. D* is the solute effective molecular diffusion coefficient, defined as:


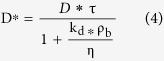


where τ is the metric tortuosity (Table 3), ρ_b_ is the density and K_d_ is the distribution coefficient. This analytical solution was obtained from Fick’s first law of diffusion. It is effective at the interface between low- and high-permeability porous media. The release of fluorescein from the low-permeability lenses into the bulk is fundamentally based on the basic mechanisms of transport therefore mainly on Back Diffusion; thus, in the proposed analytical solution, the distribution coefficient (kd) was considered equal to zero because it can influence only the lower or greater availability of the entrapped substance. As shown in Fig. 11a, the fit between the numerical and experimental trends is rather good (E = 0.99). Over the last four days, I.A. underestimates the medium diffusive flow values, mainly due to the low fluorescein concentrations.

From Fig. 11b, it is confirmed that with the same contact time, the solute intrusion into the different lenses is different, depending on the grain size: the smaller the grain size is, the larger the amount of solute that will be stored is. The figure reports the evaluated amount for the single lenses vs time; it is possible to observe both the larger concentration (made dimensionless with respect to the solute concentration in the bulk fluid) stored in the thinner material and the higher value (compared with the other lenses) contained after 14 days in the same lens. This evidence therefore has strong implications on the remediation techniques, which that must be chosen properly by carefully considering the soil texture.

## Conclusion

In this paper, a laboratory experiment to study the release of contaminant from low-permeability zones into groundwater flow is presented. Experimental studies can be extremely useful for a better interpretation of the process of redistribution from low-permeability lenses; furthermore, the results can be useful for implementing a numerical model that can provide information for both the process and the eventual restoration of the aquifer. As a result, the information on the fluxes derived from lower permeability lenses and the parameterization of the main processes encountered can help clarify the involved processes.

For these reasons, the procedure implemented in the experimental tests and the I.A. used to calculate the concentrations are found to be suitable for reproducing the processes by means of an experimental approach at the laboratory scale.

Some of the results obtained could be considered obvious, e.g., the larger storage of contaminants in the lower permeability areas; however, the possibility offered in the procedure of relating the concentration values (and validating the results through mass balance control) inside the aquifer to the dimensions and texture of the involved layer can give quantitative answers to several questions that cannot be obtained directly from qualitative considerations.

The method used to calculate the medium diffusive flux from the lenses as a function of time confirmed the higher storage capability of smaller and medium size lenses. Thus, based on these results, the lower the average grain dimension of the formation is, the higher the flux is and the longer the time is over which the values of the released concentration can constitute a source of contamination. The obtained exponential decay of the flux in time (R^2^ between 0.92 and 0.93) can surely be considered an interesting indication of the times involved in this type of contamination, which is caused by a solute plume encountering a lower permeability zone. The possibility of obtaining a law that can either indicate the residual times in the re-diffusion process or validate the reported analytical relation derived from Yang *et al*.^10^ is a good basis for further studies. Notably, the long-term application of a technology that can enhance the mobilization of the solute (e.g., aggressive pumping to reach a fast depletion of the source) may not be as effective over long periods due to semi-equivalence between the fluxes generated from the advective motion of the water and the molecular diffusion at the interface between the water and the low-permeability lenses.

The first results from the reported experiments can give information on the specific phenomena that accomplish the “Back Diffusion” process. The main evidence is that the contamination tail can’t be remediated just by a mechanical enhance of the flow velocity in the bulk media, furthermore the evidence on the importance of the diffusion transport can suggest that the improve in the temperature could bring a faster dissolution from the lens to the bulk medium. Further investigations that account for different groundwater velocities, temperature variations and spill scenarios are necessary prior to establishing a generalization of the results and the subsequent application of the empirical laws derived. Because of the enormous difficulties in conducting field tests, laboratory-scale experiments play an important role in defining suitable mathematical relationships for the correct treatment of these contamination scenarios.

## Additional Information

**How to cite this article**: Tatti, F. *et al*. Image analysis procedure for studying Back-Diffusion phenomena from low-permeability layers in laboratory tests. *Sci. Rep.* **6**, 30400; doi: 10.1038/srep30400 (2016).

## Figures and Tables

**Table 1:** Parameters for low- and high-permeability media.

	Materials	γs (kg/dm^3^)	Medium Grain Size (μm)	Porosity
Sand	Quartz	2.65	700	0.40
Lens 1	Quartz Flour	2.65	15	0.40
Lens 2	Quartz Flour	2.65	12	0.42
Lens 3	Sodium Bentonite	2.35	1.4	0.60

**Table 2:** Coefficient of efficiency (E) for observed concentration and concentrations obtained by I.A.

Day	Coefficient of efficiency (E) for Blue color	Coefficient of efficiency (E) for Green color
1	0.9	−0.4
2	−1.7	0.8
3	—	0.9
4	—	0.9
5	—	0.3

**Table 3:** Parameters of fluorescein and of lens 1 for the analytical solution proposed by Yang *et al*. 10.

Parameters	Values	Units	Notes
τ	0.6	—	Chapman *et al*.^8^
D	13.0E-10	m^2^/s	Chapman *et al*.^8^
